# Finding wolf homesites: improving the efficacy of howl surveys to study wolves

**DOI:** 10.7717/peerj.5629

**Published:** 2018-09-28

**Authors:** Thomas D. Gable, Steve K. Windels, Joseph K. Bump

**Affiliations:** 1 Department of Fisheries, Wildlife, and Conservation Biology, University of Minnesota, St. Paul, MN, United States of America; 2 Voyageurs National Park, International Falls, MN, United States of America

**Keywords:** Voyageurs National Park, *Canis lupus*, Observer error, Predator, Acoustic surveys, Triangulation, Acoustic triangulation, Rendezvous site, Wolf den, Wolf pups

## Abstract

Locating wolf (*Canis lupus*) homesites is valuable for understanding the foraging behavior, population dynamics, and reproductive ecology of wolves during summer. During this period wolf pack members (adults and pups) readily respond to simulated wolf howls (i.e., howl surveys), which allows researchers to estimate the location of the homesite via triangulation. Confirming the actual locations of homesites via ground truthing is labor intensive because of the error surrounding estimated locations. Our objectives were (1) to quantify observer error during howl surveys and compare amongst experience levels, (2) provide a simple method for locating homesites in the field by incorporating observer error, and (3) further document the value of this method for monitoring wolf packs throughout the summer. We located 17 homesites by howl surveys during 2015–2017 in the Greater Voyageurs Ecosystem, Minnesota, USA. Of 62 bearings taken by observers during howl surveys, bearings erred by an average of 7.6° ± 6.3° (SD). There was no difference in observer error between novice and experienced observers. A simple way to increase efficiency when searching for homesites is to search concentric areas (bands) based on estimated observer error, specifically by: (1) adding ±10° error bands around howl survey bearings when ≥3 bearings can be obtained, (2) ±10° and ±20° error bands when 2 bearings are obtained, and (3) ±10° and ±26° error bands when 1 bearing is obtained. By incorporating observer error and understanding how frequently and how far wolves move homesites, it is possible to monitor wolf packs and confirm most, if not all, homesites used by a pack from at least June until August without having a collared individual in a pack.

## Introduction

Gray wolves (*Canis lupus*) are elusive predators that are challenging to study during the snow-free season, especially in densely forested habitats with limited visibility (Blanco & Cortes, 2012). In late winter to late spring (∼ late March–early May), depending on latitude, breeding female wolves will localize at a natal den and produce a litter of pups. Wolves keep pups in dens initially but eventually transition to using rendezvous sites—which act as above-ground den sites for the pups—by late spring or early summer once pups are ∼5–6 weeks old (Fuller, 1989; Mills, Patterson & Murray, 2008). During the spring and summer, these homesites (i.e., den and rendezvous sites) become the focal point of wolf pack activity with pack members leaving and returning between foraging trips (Demma & Mech, 2009). Through summer, adult wolves spend less time at homesites as the pups become more mobile and independent (Potvin, Peterson & Vucetich, 2004; Ruprecht et al., 2012). By late summer or early fall, the pack stops using homesites as the pups are almost full grown and capable of traveling nomadically around their territory with other pack members (Mills, Patterson & Murray, 2008).

Because wolf homesites are often rich with wolf scats, wolf hair, and prey remains (Joslin, 1967), locating wolf homesites is valuable for non-invasive collection of biological samples that can be used to understand the foraging behavior, population dynamics, and reproductive ecology of wolves. Summer wolf diet studies are often based on scats collected at homesites (Fuller, 1989; Sidorovich, Tikhomirova & Jedrzejewska, 2003; Hayes, Baer & Clarkson, 2016; Gable, Windels & Bruggink, 2017; Ciucci et al., 2018). Genetic analysis of scats and hair samples from homesites is a useful approach to understand wolf pack size, recruitment, pup survival, and phylogeny (Stenglein et al., 2010; Stenglein et al., 2011; Adams et al., 2011; Stansbury et al., 2016; Ausband et al., 2017). Identifying homesite locations is also important for determining the factors that influence where wolves establish homesites (Unger et al., 2009; Kaartinen, Luoto & Kojola, 2010; Klaczek, Johnson & Cluff, 2015; Jacobs & Ausband, 2018), why they abandon them (Ausband et al., 2016), and how site selection affects pup survival (Benson, Mills & Patterson, 2015). As wolves continue to expand their global range and inhabit more human-dominated landscapes understanding how human-related risk and disturbance affect homesite selection (Sazatornil et al., 2016) and occupancy (Argue, Mills & Patterson, 2008) provides valuable information for wolf conservation (Mech, 2017).

Howl surveys are a relatively non-invasive tool (Stenglein et al., 2010; Leblond, Dussault & St-Laurent, 2017) to identify wolf pack homesite locations during the summer and are generally used when no wolves in a specific pack are fitted with a Very High Frequency (VHF) or Global Positioning Systems (GPS) collar (Harrington & Mech, 1982). During the homesite period—specifically June to August—wolf pack members (adults and pups) readily respond to simulated wolf howls (i.e., howl surveys), which allows researchers to estimate the location of a homesite via triangulation (Joslin, 1967). Typically, howl surveys are used to locate homesites by: (1) simulating wolf howls around dawn or dusk when there is little wind to impede the hearing of observers, (2) estimating the bearing of the wolf response, often from three or more different locations so that the site can be triangulated, and (3) attempting to find the homesite immediately after hearing the response (Ausband et al., 2010) or in the following days or weeks (Joslin, 1967; Gable, Windels & Bruggink, 2017).

Even after triangulation, homesites can be difficult to find in the field, often requiring several hours of searching (Pimlott, Shannon & Kolenosky, 1969; T Gable, pers. obs., 2017). Much of this difficulty is related to the error around bearings recorded during howl surveys (i.e., how many degrees an observer’s bearing is from the true bearing of a homesite). This is especially true when homesites are outside of a triangulated area or when triangulation of homesites cannot be achieved (i.e., when only one or two bearings are recorded during a survey). Reducing the time spent searching for homesites would improve the utility of howl surveys. Given this, our objectives were (1) to quantify observer error during howl surveys and examine whether this error is related to observer experience, (2) to propose an improved, simple method for locating homesites by incorporating observer error, and (3) further document the value of howl surveys for monitoring wolf packs throughout the summer. We expected, based on substantial howl survey experience, a positive quadratic relationship between observer error and the distance between the observer and the homesite. That is, observer error would be largest when observers were close to and then far away from homesites, due to the volume and reverberation dynamics of wolf howls.

### Study area

Our study occurred in the Greater Voyageurs Ecosystem (GVE) which includes Voyageurs National Park and the area of the north-central Kabetogama State Forest, Minnesota (48°33′N, 92°90′W) that is southerly adjacent to Voyageurs National Park. This area is part of the Laurentian Mixed Forest Province and on the southern edge of the boreal forest (Bailey, 1980). Voyageurs National Park is predominantly composed of mixed forest habitats interspersed with wetlands such as beaver ponds and black spruce (*Picea mariana*) bogs (Gable & Windels, 2017). The Kabetogama State Forest, in contrast, is actively logged which results in a mosaic of clear cuts, young aspen (*Populus* spp.) stands, mature deciduous-coniferous stands, and wetlands (Gable et al., 2018). Logging road and all-terrain vehicle trails are common throughout the Kabetogama State Forest. White-tailed deer are common (pre-fawn densities 2–4 deer/km^2^; Gable, Windels & Olson, 2017) throughout the GVE and moose (*Alces americanus*) relatively uncommon (<0.15 moose/km^2^ in the interior of Voyageurs National Park), especially in the Kabetogama State Forest (<0.05 moose/km^2^; Windels & Olson, 2017). The area supports a dense beaver population with densities generally ranging from 0.47 lodges/km^2^ to >1 lodge/km^2^ (Johnston & Windels, 2015; Gable & Windels, 2017). Summer wolf densities in the area are ∼4–6 wolves/100 km^2^ with average summer home ranges of 116 km^2^ (Gable, 2016). White-tailed deer (*Odocoileus virginianus*) and black bear (*Ursus americana*) hunting, and fur trapping are popular recreational activities in the Kabetogama State Forest but these activities are not permitted in Voyageurs National Park. Hunting and trapping of wolves was prohibited in Minnesota during our study as wolves were considered a threatened species and protected under the Endangered Species Act (Mech, 2017).

### Methods

### Howl surveys and locating homesites

We used howl surveys during 2015–2017, in conjunction with GPS-collared wolves to locate wolf homesites to gather data on the reproductive ecology of wolves during the summer in the GVE and to collect biological samples (scat, hair samples, and prey remains). Howl surveys were done in the summer (early June until late August) to locate homesites from packs that did not have at least one wolf fitted with a GPS collar (all capture and handling of wolves was approved by the National Park Service’s Institutional Animal Care and Use Committee [protocol no.: MWR_VOYA_WINDELS_ WOLF]). We only surveyed on evenings when there was no precipitation and wind speeds were minimal (<5–7 kph) (Harrington & Mech, 1982; Fuller & Sampson, 1988). Our surveys started at 20:00–21:00 and lasted 4–9 hr. If survey conditions deteriorated in the field, we stopped the survey. During surveys, 1 observer simulated wolf howls by howling three times with 15–30 s between each howl (Fuller & Sampson, 1988). The observer would repeat this three times with 2–3 min between sets of howls, or until a response was heard by the 1–3 observers (Harrington & Mech, 1983). We commonly had individual wolves respond during howl surveys but only considered a response to come from a homesite if we heard pups howling. Pup howls are easily distinguishable from adults in the summer based on pitch (Palacios et al., 2017).

When wolves responded, observer(s) used standard handheld compasses and estimated, to the nearest degree, the bearing of the response. Standard handheld compasses are not as precise as more advanced compass types (e.g., mirror compass). However, we believed basing error estimates on standard handheld compasses would be more useful for other researchers because handheld compasses are easily used in nighttime conditions (i.e., when howl surveys are often conducted), inexpensive, and are already standard field equipment for managers and researchers.

Because we wanted three bearings from different locations to triangulate a homesite, we would simulate a howl, elicit a response, record a bearing, move to a different location, and repeat until we recorded 3 bearings. When conducting surveys in more accessible areas (i.e., areas with a network of drivable logging roads and easily walkable ATV trails), we would try to have multiple observers separated by 0.2–5 km so that we could get several bearings from a single howling response, thus reducing survey time. We surveyed 1–2 packs per evening depending on accessibility and how quickly packs could be located. During some surveys we could only record 1–2 bearings but still searched for and located homesites using the general approach described below.

When we searched for homesites, we uploaded the howl survey results (i.e., the observer bearings [as lines] and, when applicable, the triangulated area likely containing the homesite) from GoogleEarth (Google, California, USA) onto a handheld GPS device (GPSMAP 64s, Garmin, Kansas, USA). We did not attempt to search for triangulated homesites until wolves abandoned the site or it was the end of a calendar month (see below). Once a site was identified during surveys, we monitored the site via howl surveys every 5–10 days until the site was abandoned. To do this, we howled from the location at which we had previously heard a response. If we heard a new response, we took a bearing to determine whether the pack was occupying the same homesite. If we did not get a response, we assumed the site had been abandoned and attempted to locate the new site as described above.

**Figure 1:**
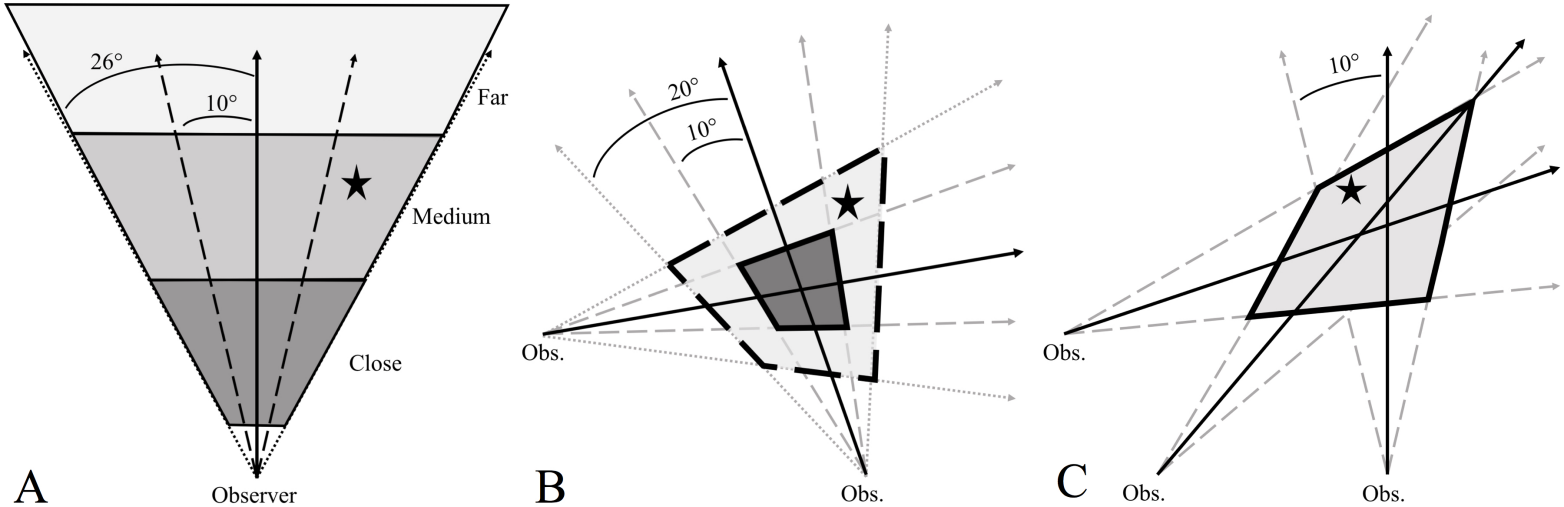
Examples of how incorporating observer error in howl surveys helps focus the search area, and presumably the time spent searching for, wolf homesites (black star). Observer error is the difference (in degrees) between an observer’s estimated bearing (solid black arrows) of a wolf response and the true bearing of the homesite. When one bearing is obtained (A), the observer estimates how close the response is (close, medium, far) and then incorporates ±10° and ±26° error (dashed black lines) around their bearing to determine an area that likely contains the homesite. When two bearings are obtained (B), incorporating ±10° and ±20° error around observer bearings creates two polygons: a smaller 10°-error polygon (dark gray, solid outline) that will commonly contain the homesite and a larger 20° -polygon (light gray, dashed outline) that almost certainly contains the homesite. When at least three bearings are obtained (C), incorporating ±10° error around observer bearings will create an error polygon that almost certainly contains the site.

We searched for homesites once the site was abandoned to document the site and collect biological samples. We tried not to visit homesites until after they were abandoned because wolves generally left sites once we visited and established new homesites elsewhere. However, per the methods of a multi-year wolf diet study in the GVE (Gable, Windels & Bruggink, 2017), we did visit occupied homesites at the end of the calendar month. To locate homesites, a crew of 2–3 individuals started by searching the search polygon (either a triangulated area [when three bearings were recorded] or polygon [when >3 bearing were recorded]) identified as likely containing the homesite (Fig. 1) using handheld GPS devices to ensure we thoroughly searched the entire search polygon. Because GVE wolves commonly establish homesites along habitat edges, particularly edges of wetlands and bogs (Windels, 2017; T Gable, 2017, unpublished data), we typically searched these habitat features within the search polygon first to expedite locating the site. Homesites, which are generally <15 ha in size (T Gable, 2018, unpublished data), have a central or focal area, where most wolf activity occurs (Stansbury et al., 2014). These focal areas are typified by trampled and depressed vegetation, an abundance of wolf scats, and multiple well-worn trails leading to and from the area. If the site was not found in the search polygon, we expanded our search to the area outside of the search polygon and used a back-and-forth search pattern to systematically search the area until the site was found. Once a homesite was located, we searched the area intensively following all wolf trails and noting all wolf sign to identify the focal area of the site. We recorded a GPS waypoint in the center of focal area and used this location for all subsequent analyses.

### Analysis

We wanted to develop a simple, efficient method for locating wolf homesites from howl surveys when one, two or ≥3 bearings are recorded. We determined that by quantifying how much observer bearings erred from the true bearing to the focal point of the homesite, we could develop simple guidelines to aid in locating homesites in the field. To do so, we used an observer’s location and the confirmed location of the homesite via ground-truthing to compare the observer’s estimated bearing (i.e., where the observer thought the response was coming from) to the actual bearing of the homesite relative to the observer’s location. We assumed that responding wolves were at the focal point of the homesite but we are aware this was not always the case, as wolves spend time in different areas in and around homesites (Ruprecht et al., 2012). Therefore, we did not consider observer error to strictly be the acoustic error (i.e., the difference between where an observer thinks a response is emanating and where the response is actually originating). Rather, we considered observer error to be the absolute value of the true bearing to the focal area of the homesite minus the observer’s bearing. That is, observer error was the combination of the acoustic error plus the unknown difference between the location of the howling wolves and the focal area of the homesite. Quantifying error this way removes the uncertainty of where responding wolves might be relative to the homesite and thus provides a more practical method to estimate the location of, and then find, homesites.

We compared experienced observers to novice observers to test for differences in observer error based on observer experience. Observers were considered experts if they had >3 months experience conducting howl surveys and novices if they had <3 months experience. The 3-month cutoff was chosen because this is commonly the minimum duration of seasonal field positions conducting fieldwork on wolves. Thus individuals who had >3 months experience had worked at least one field technician position in which howl surveys were a large portion of their duties, and those with <3 months experience had not. To compare results, we calculated the mean error for each observer (i.e., the sample unit) and used an Independent Samples *T*-Test. We used a percentile bootstrapping approach to estimate 95% confidence intervals for mean expert and novice observer error (Gable et al., 2018). We used quadratic regression to test if there was a relationship between observer error and the distance of the observer from the homesite.

## Results

### Howl surveys

In total, we conducted 21 successful howl surveys. We located 17 homesites via howl surveys during June–August. In the other four instances, we successfully re-surveyed (i.e., elicited a response) an occupied site 5-10 days after initially locating the site. There was only one instance where we triangulated a homesite but were unable to locate the site. We suspect we triangulated the pack while they were moving homesites because we did not find any sign of wolves or a homesite in 4 field days (24 hr) of searching.

**Figure 2:**
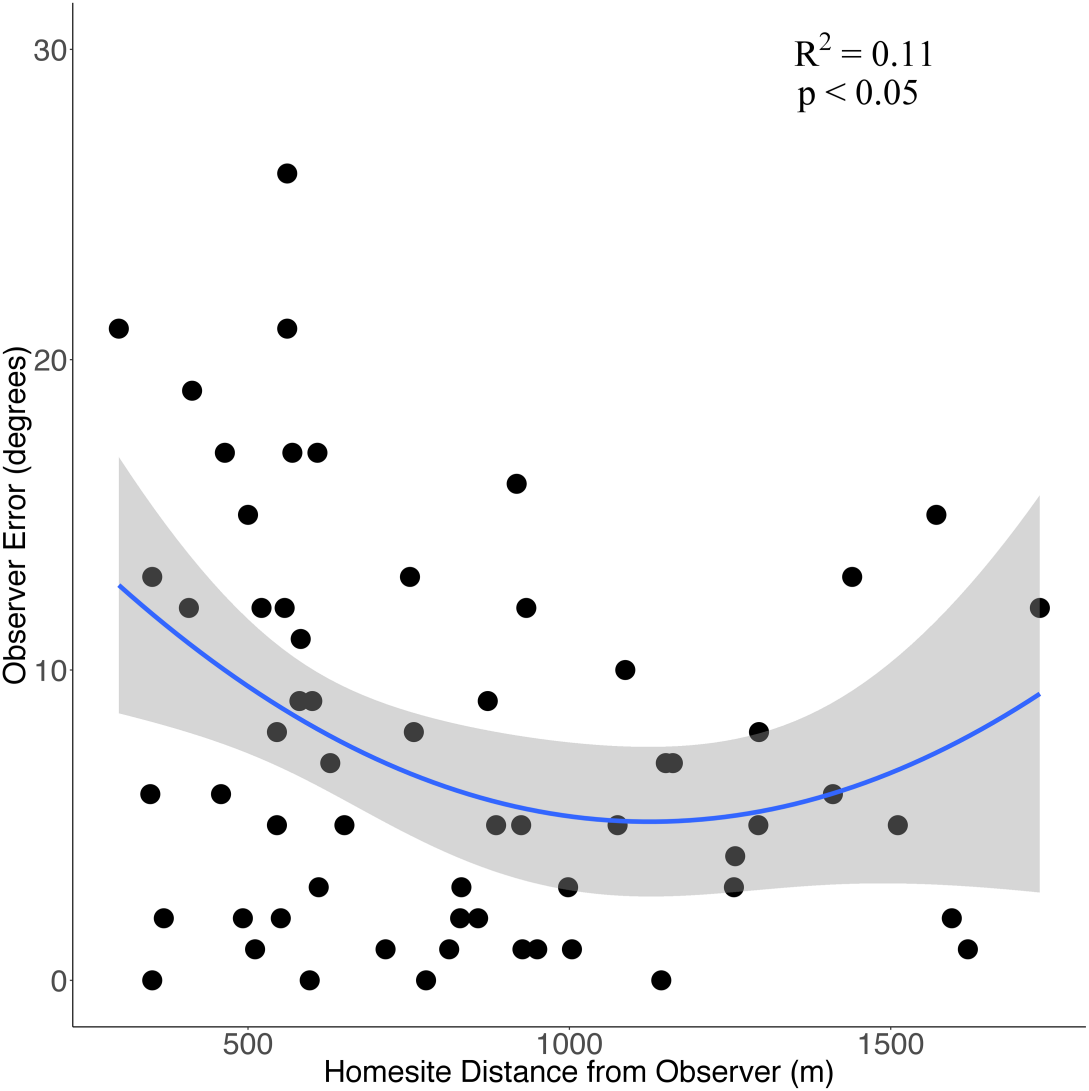
The relationship between observer error and distance (m) from homesites when conducting howl surveys of wolf homesites during June-August in the Greater Voyageurs Ecosystem. Observer error is the difference (in degrees) between an observer’s estimated bearing of a wolf response and the true bearing of the homesite. The solid blue line represents the quadratic relationship between observer error and distance from homesites and the shaded region represents the 95% confidence interval.

Eight observers (three experienced observers, five novice) recorded a total of 62 bearings from these 21 successful surveys. There was no significant difference in mean error between novice (7.9° ± 5.1° [SD]) or experienced observers (7.6° ± 2.0°; *P* = 0.93) so we pooled observer data to compare how observer error changed in relation to distance from homesites. On average, observer bearings were off by 7.6° ± 6.3° (SD; range = 0°–26°; 95% confidence interval: 6.2°–9.2°) with 69% of bearings incorrect by <10°, 87% of bearings by <15° and 95% by <20°. There was a positive but weak quadratic relationship between observer error and observer distance (in meters) from homesites (error = 19.25–0.0252*distance + 0.0000112*distance^2^, *R*^2^ = 0.11, *P* < 0.05, Fig. 2).

Locating homesites first detected via howl surveys was often challenging and time consuming. Excluding the failed attempt to locate a homesite (described above), we were unable to triangulate homesites during 35% (6/17) of howl surveys (we attempted triangulation during the initial surveys [*n* = 17] but not re-surveys [*n* = 4]) either because we could not get >2 bearings (i.e., the wolves did not respond multiple times; occurred during 2/17 successful surveys) or because our bearings did not converge to form a triangle (occurred during 4/17 successful surveys). However, even when we were able to triangulate a response (*n* = 12), 42% (*n* = 5) of homesites were outside of the triangulated area.

## Discussion

Incorporating estimates of observer error into howl survey bearings focuses the search area and presumably the time spent searching for homesites. We frequently were not able to triangulate homesites and when we did the homesite was commonly not in the triangulated area. We suggest a simple way to increase search efficiency is to add ±10° error around survey bearings when ≥3 bearings can be obtained. The ±10° error is greater than the mean observer error (±8°) but is more useful because the ‘bands’ (see Fig. 1A) created by including ±10° error around a single bearing would contain a homesite 69% of the time compared to 57% of the time when using mean observer error. By drawing or digitizing the ±10° error for multiple bearings, an ‘error polygon’ can be created from where the outermost ‘error bearings’ overlap, thus making a polygon that almost certainly contains the homesite location (Fig. 1). For example, 100% of homesites for which we obtained at least 3 bearings would have been within the ±10° error polygon, even though observer error was >10° for some bearings. Even when incorporating observer error, the error polygon can be sizable, depending on the distance and direction of the site from the observers during the howl survey. When two bearings are obtained, we suggest adding error of ±10° and ±20° (95% of observer errors were ≤20°). Doing so will create 2 polygons, with the small polygon (the ‘search polygon’) having a high probability of containing the site and the large polygon (the ‘error polygon’) almost certainly containing the site (probability of getting 2 bearings with errors >20° is 0.0025 [0.05^2^]).

We suggest searching the triangulated area (if triangulation was achieved) or search polygon first as many homesites will likely be in this area (e.g., when triangulation was achieved, 58% of sites were in triangulated area) and then, if the homesite cannot be found, expanding the search to the error polygon. Search time can also be minimized by searching landscape features where there is a high probability of homesite occurrence (Ausband et al., 2010). For instance, in habitats similar to northern Minnesota or central Ontario, searching edges of wetlands, bogs and beaver meadows, where wolves commonly establish homesites, can greatly expedite finding homesites (Joslin, 1967; Benson, Mills & Patterson, 2015; Windels, 2017; T Gable, 2017, pers. obs).

One of the benefits of quantifying observer error during howl surveys is that it allows homesites to be located even if only a single response was heard and one bearing obtained (Fig. 1). In such instances we suggest adding an error of both ±10° and ±26° (maximum observer error). Doing so will create two triangles that radiate from where the observer heard the response. These triangles can then be searched based on perceived proximity of the howl response. With practice it becomes easy to estimate the general distance (e.g., close, medium distance, or far away) of homesites from the sound of a response. In our study area, close or loud responses were generally between 300–700 m from the observer, medium responses between 800–1,200 m, and distant responses >1,200 m (T Gable, 2017, pers. obs). The ±10° error triangle should be searched first as most of the time this should contain the site (69% of single bearings with ±10° error would contain the site). However, depending on how far the site is to the observer, the area to be searched can be large (even with ±10° error). Thus, in many cases it would likely be advantageous to get another bearing to decrease the search area and find the homesite more efficiently.

The distance of the observer from the homesite did affect observer error, though it only explained 11% of the variation in observer error. Observer error appears to be largest when observers are close to and far away from homesites with the smallest error occurring when the observer is a moderate distance (∼1.0–1.3 km) away (Fig. 2). It may seem counterintuitive for error to be higher when the site is close to observers but this is consistent with our observations in the field when searching for homesites. Observers generally felt more confident about their bearings at close distances because the howls were loud and clear. However, proximity was not a reliable indicator of accuracy and it took as long to locate homesites that were close as those far away. We think it can be difficult to pinpoint the direction of a howl at close distances because of the volume and echo of the response. Nonetheless, observer distance from the homesites should not be problematic when finding homesites from howl surveys if observer error is incorporated as described above. We only recorded responses ≤1,732 m away so observer error could be substantially higher at greater distances.

Many factors affect when and how frequently wolves howl and respond to simulated howls (Harrington & Mech, 1978; Nowak et al., 2006; Mcintyre et al., 2017). Although the June to August period appears to be when wolves are most likely to respond to simulated howls (Joslin, 1967; Nowak et al., 2006), sometimes wolves simply will not respond (Harrington & Mech, 1982). We observed pack-specific differences in likelihood to respond. We had 3 packs that responded to every simulated howl (*n* = 27) near their homesite (provided we waited ∼15 mins from when the wolves last howled; Harrington & Mech, 1983). Thus, we had little issue monitoring these packs throughout the summer and getting an adequate number of responses to triangulate their homesites. However, we had two packs that were unique. The first was a newly established pack (two adult wolves and pups). During June–July, the pups would often respond to the first simulated howl but would not respond to subsequent howls (the adults were seldom at the homesite during our surveys though we commonly heard the adults howling within 3 km of the site). On a few nights, though, the pups would not respond to any howls. The second was a remote pack we surveyed on 27 July, 2017. We elicited a response at about 21:00 but then spent several hours, to no avail, attempting to get a second response. Finally, 7 hr later at 4:00 on 28 July the pack responded. We returned the next evening to get another response and a 3rd bearing but could not elicit a response.

## Conclusions

Based on this work, we contend that howl surveys are a useful tool to locate most, if not all, homesites used by a pack from at least June until August without having a VHF or GPS-collared individual in a pack. The most challenging aspect of locating all wolf homesites throughout the summer via howl surveys is initially finding a pack’s homesite via howl surveys. Once a site is found, it can be re-surveyed every 5 days to 2 weeks, depending on the time of year and study area, until the pack moves. After the pack moves, the abandoned homesite can be located in an efficient manner by incorporating estimates of observer error as described above, and the newly established homesite identified by conducting howl surveys at strategic locations that cover the entire area of possible new homesite locations (based on the distance pack’s move homesites; for more information see Mills, Patterson & Murray, 2008 and Ausband et al., 2016). Strategic howling locations should take into account the limitations of human auditory abilities. Though some have suggested observers can hear wolf responses 3.2–5 km away (Harrington & Mech, 1982), most evidence suggests that observers can only hear wolf responses from <2–2.5 km (Fuller & Sampson, 1988; Nowak et al., 2006). The lead author (TDG) has clearly heard simulated howls from observers 2.2 km away on a calm night.

Our approach can easily be implemented in areas with adequate road and trail access but will be more challenging and time-intensive in remote field settings. Further, because some packs will not respond, at times, to simulated howls means that some areas or packs will need to be re-surveyed multiple times to locate homesites. In a few instances, we could not find, during our initial survey, the new homesite of a pack that moved but because we knew the plausible distance the pack could have moved (using data from the literature Mills, Patterson & Murray, 2008; Ausband et al., 2016), we found the homesite when we re-surveyed the area a day later. We only conducted howl surveys during June-August (when wolves are using homesites and are most likely to respond Joslin, 1967; Harrington & Mech, 1982) but we suggest, based on our hearing pups howling with adults at late May homesites, that howl surveys at the end of May could be a promising method to help find May homesites.

Ultimately, this approach provides an effective, though at times field intensive, method to study and monitor wolves throughout the summer. Granted, the field effort necessary for this approach is likely no more time intensive than capturing, collaring, and subsequently monitoring wolves. This approach will never be better or more effective than a GPS-collared wolf in a pack, but it provides a method to collect substantial amounts of data and monitor packs when non-invasive methods are desired, or capture and collaring is unsuccessful or not possible. For example, in April and May 2017, we unsuccessfully attempted to capture and collar at least one wolf from two packs using foothold traps. However, by using our approach we were able to locate every homesite (10 sites) from these two uncollared packs during June–August 2017. In total, this allowed us to collect 260 scats, prey remains, and several hair samples, confirm both packs had multiple pups, and get a minimum pup count visually for one pack. Thus, we have confirmed that this approach can be a valuable tool for researchers who want to study specific wolf packs throughout the summer.

##  Supplemental Information

10.7717/peerj.5629/supp-1 Data S1 Howl survey data Howl survey data from Voyageurs National Park from 2015–2017.

10.7717/peerj.5629/supp-2 Data S2 R code for statistical analysis

10.7717/peerj.5629/supp-3 Data S3 Statistical analysis for howl survey and homesite data

## Additional Information and Declarations

### Data Availability

The following information was supplied regarding data availability: The raw data are provided in the Supplemental Files.
