# Supplementary material for: Finding wolf homesites: improving the efficacy of howl surveys to study wolves
Source: PeerJ. 2018 Sep 28;6:e5629. doi: 10.7717/peerj.5629 (PMC6166618; doi:10.7717/peerj.5629)
Supplement: Data S3 [file peerj-06-5629-s003.html]

HowlSurvey\_DataAnalysis.R


# HowlSurvey\_DataAnalysis.R

#### *thomasgable*

#### *Sun Aug 12 08:03:20 2018*

```
library(mosaic)
library(dplyr)
library(ggplot2)
library(scales)
```

## HOWL SURVEY DATA

```
howl<-read.csv(file="2017howlsurvey_data.csv")
howl$Hsdistance<-as.numeric(howl$Hsdistance)
```

Comparing observer error across experience levels (novice vs. experienced)

```
obsErr<-favstats(bearDiff~observer,data=howl)
obsErr$skill<-c("e","n","n","n","e","n","n","e") #e=experience,n=novice
nov<-obsErr[obsErr$skill=="n",] #filtering out experienced observer
mean(nov$mean) #mean error for novice observers
```

```
## [1] 7.655556
```

```
sd(nov$mean) #standard deviation for novice observer error
```

```
## [1] 5.062632
```

```
exp<-obsErr[obsErr$skill=="e",]
mean(exp$mean) #mean error for experienced observers
```

```
## [1] 7.87963
```

```
sd(exp$mean) #standard deviation for experienced observer error
```

```
## [1] 2.03373
```

Independent Samples T-Test comparing experienced vs. novice observers

```
t.test(mean~skill, data=obsErr)
```

```
## mean ~ skill
```

```
## 
##  Welch Two Sample t-test
## 
## data:  mean by skill
## t = 0.087857, df = 5.6269, p-value = 0.933
## alternative hypothesis: true difference in means is not equal to 0
## 95 percent confidence interval:
##  -6.118308  6.566456
## sample estimates:
## mean in group e mean in group n 
##        7.879630        7.655556
```

Because there was no difference between experience levels, we combined observers for subsequent analysis

```
mean(obsErr$mean) #mean observer error
```

```
## [1] 7.739583
```

```
sd(~bearDiff,data=howl) #standard deviation of observer error
```

```
## [1] 6.269712
```

Percentile Bootstrap for 95% Confidence Interval for Observer Error

```
bootDist<-do(1000)*{
  bootSamp<-resample(howl$bearDiff)
  mean(bootSamp)
}
quantile(bootDist$result, probs=c(0.025,0.975)) #95% Confidence Interval
```

```
##     2.5%    97.5% 
## 6.049180 9.196721
```

Quadratic regression to assess the relationship between observer error and distance from homesites

```
distModel<-lm(bearDiff~Hsdistance+I(Hsdistance^2),data=howl) 
summary(distModel)
```

```
## 
## Call:
## lm(formula = bearDiff ~ Hsdistance + I(Hsdistance^2), data = howl)
## 
## Residuals:
##      Min       1Q   Median       3Q      Max 
## -11.8050  -4.4531  -0.5578   3.1797  17.3351 
## 
## Coefficients:
##                   Estimate Std. Error t value Pr(>|t|)    
## (Intercept)      1.925e+01  4.641e+00   4.149  0.00011 ***
## Hsdistance      -2.515e-02  1.080e-02  -2.328  0.02342 *  
## I(Hsdistance^2)  1.118e-05  5.529e-06   2.022  0.04781 *  
## ---
## Signif. codes:  0 '***' 0.001 '**' 0.01 '*' 0.05 '.' 0.1 ' ' 1
## 
## Residual standard error: 6.012 on 58 degrees of freedom
## Multiple R-squared:  0.1112, Adjusted R-squared:  0.08057 
## F-statistic: 3.629 on 2 and 58 DF,  p-value: 0.03274
```

```
par(mfrow=c(2,2))
plot(distModel) #'Checking diagnostics. Meets assumption of regression pretty well.
```

```
par(mfrow=c(1,1))
```

Figure showing relationship between observer error and distance from homesites

```
Fig2<-ggplot(howl, aes(x=Hsdistance, y=bearDiff))+geom_point(size=8)+
  geom_smooth(method="lm", formula=y~x+I(x^2),se=T,size=2)+
  theme_classic()+scale_x_continuous(name="Homesite Distance from Observer (m)")+
  scale_y_continuous(name="Observer Error (degrees)")+
  theme(axis.text = element_text(size=28),axis.title = element_text(size=28))+
  annotate("text", x=1460, y=30, label=paste("R^2 == ", 0.11), size=12, family="Times New Roman", parse=TRUE)+ #manually added text to graph because I couldn't get R2 to display
  annotate("text", x=1450, y=28.5, label="p<0.05", size=12, family="Times New Roman", parse=TRUE) #manually adding the p=value
Fig2
```

For saving figures as png

```
png(filename="Fig2_HowlSurvey.png", #make the png file 
    type="cairo",
    units="in", 
    width=14, 
    height=14, 
    pointsize=12, 
    res=300) #dpi

Fig2

dev.off()
```

```
## quartz_off_screen 
##                 2
```
